# Inhibiting miR-22 Alleviates Cardiac Dysfunction by Regulating Sirt1 in Septic Cardiomyopathy

**DOI:** 10.3389/fcell.2021.650666

**Published:** 2021-04-01

**Authors:** Runze Wang, Yuerong Xu, Wei Zhang, Yexian Fang, Tiqun Yang, Di Zeng, Ting Wei, Jing Liu, Haijia Zhou, Yan Li, Zhan-peng Huang, Mingming Zhang

**Affiliations:** ^1^Department of Cardiology, Tangdu Hospital, The Fourth Military Medical University, Xi’an, China; ^2^Department of Hematology, Tangdu Hospital, The Fourth Military Medical University, Xi’an, China; ^3^Department of Orthodontics, School of Stomatology, The Fourth Military Medical University, Xi’an, China; ^4^Department of Cardiology, Center for Translational Medicine, The First Affiliated Hospital, Institute of Precision Medicine, Sun Yat-sen University, Guangzhou, China; ^5^NHC Key Laboratory of Assisted Circulation, Sun Yat-sen University, Guangzhou, China

**Keywords:** miR-22, autophagy, apoptosis, septic cardiomyopathy, sirt1

## Abstract

High morbidity and mortality are the most typical characteristics of septic cardiomyopathy. We aimed to reveal the role of miR-22 in septic cardiomyopathy and to explore the underlying mechanisms. miR-22 cardiac-specific knockout (miR-22^cKO^) mice and miR-22 cardiac-specific transgenic (miR-22^cOE^) mice were subjected to a cecal ligation and puncture (CLP) operation, while a sham operation was used in the control group. The echocardiogram results suggested that miR-22^cKO^ CLP mice cardiac dysfunction was alleviated. The serum LDH and CK-MB were reduced in the miR-22^cKO^ CLP mice. As expected, there was reduced apoptosis, increased autophagy and alleviated mitochondrial dysfunction in the miR-22^cKO^ CLP mice, while it had contrary role in the miR-22^cOE^ group. Inhibiting miR-22 promoted autophagy by increasing the LC3II/GAPDH ratio and decreasing the p62 level. Additionally, culturing primary cardiomyocytes with lipopolysaccharide (LPS) simulated sepsis-induced cardiomyopathy *in vitro*. Inhibiting miR-22 promoted autophagic flux confirmed by an increased LC3II/GAPDH ratio and reduced p62 protein level under bafilomycin A1 conditions. Knocking out miR-22 may exert a cardioprotective effect on sepsis by increasing autophagy and decreasing apoptosis via sirt1. Our results revealed that targeting miR-22 may become a new strategy for septic cardiomyopathy treatment.

## Introduction

Sepsis is a serious systemic inflammatory reaction with high mortality caused by bacterial infection. The main causes of sepsis include serious complication of severe infection, severe trauma, and major surgery. Further development of this condition can lead to septic shock, serious damage to many organs and finally death (Semeraro et al., 2012; Kobashi et al., 2013; Martensson and Bellomo, 2015; Wagner et al., 2015). Hence, sepsis is a serious disease that endangers human health.

MicroRNAs are a class of small non-coding RNAs composed of 19–24 nucleotides that can combine with the 3’ untranslated region (3’-UTR) of mRNA and cause mRNA degradation or inhibit mRNA translation. Numerous studies have reported that miRNAs play various roles in regulating inflammation, apoptosis, necrosis and autophagy in myocardial injury (Ambros, 2004; Condorelli et al., 2014; Pan et al., 2019). microRNA-22 (miR-22) is reported to participate in some heart diseases. It has reported that miR-22 is the most abundant miRNA expressed in heart (Condorelli et al., 2014). It has been confirmed that upregulating miR-22 can result in myocardial ischemia-reperfusion (I/R) injury by targeting mitochondria (Du et al., 2016). miR-22 can also suppress myocardial fibrosis by targeting TGFβR I (Hong et al., 2016) and promote heart failure by inhibiting PPAR/ERR-nuclear hormone receptor transcription (Gurha et al., 2013). Moreover, miR-22 participates in cardiac hypertrophy and remodeling (Huang et al., 2013).

Autophagy, an evolutionarily conserved process, can degrade cytosolic components to maintain cellular homeostasis. This process mainly degrades long-lived proteins, lipids and damaged organelles (Yao et al., 2020). It has reported that autophagy participates in the development of heart diseases, such as atherosclerosis, heart failure, diabetic cardiomyopathy (DM) and I/R injury (Zhang et al., 2017, 2019; Sun et al., 2018; Pan et al., 2019). Autophagy has been reported to be upregulated in septic cardiomyopathy and dysfunction of other organs in sepsis (Hsieh et al., 2011; Yin et al., 2019). These results revealed that autophagy may become a new strategy to improve sepsis-induced cardiac dysfunction.

The mitochondria are the main energy resource of the heart and can provide abundant ATP for the heart (Cimolai et al., 2015). Septic cardiomyopathy is mostly accompanied by severe sepsis, which results in cardiac dysfunction, including diastolic and systolic dysfunction (Court et al., 2002). A recent study reported that mitochondrial dysfunction is critical in septic cardiomyopathy. The authors demonstrated that the mitochondrial dynamics proteins Drp1 and Fis1 are involved in septic cardiomyopathy (Haileselassie et al., 2019). Other studies also reported that ameliorating mitochondrial dysfunction can improve myocardial dysfunction (Vanasco et al., 2014; Joseph et al., 2017). Overall, mitigating mitochondrial dysfunction will become a strategy for the septic cardiomyopathy treatment.

Silencing information regulator 1 (sirt1) a NAD^+^-dependent deacetylase, has been reported to be involved in many cellular metabolic processes (Yang et al., 2013). Studies have shown that sirt1 exert a cardioprotective in cardiac hypertrophy and cardiac dysfunction (Li et al., 2009). Sirt1 can promote starvation-induced autophagy by deacetylating FOXO1 (Hariharan et al., 2011). In DM, sirt1 can also reduce cardiomyocyte apoptosis rate and alleviate endoplasmic reticulum stress (Guo et al., 2015). And AMPK can increase NAD^+^ further active sirt1 and finally enhanced Na^+^/K^+^ATPase to alleviate cardiac dysfunction (Witczak et al., 2008). Another study has reported sirt1 participates in attenuating high glucose-induced oxidative stress and cardiac fibrosis (Li et al., 2019). In heart failure models were established by transverse aortic constriction, taurine increased NAD^+^/NADH ratio, promoted the expression of sirt1 and suppressed p53 acetylation (Liu et al., 2020). Sirt1 regulates many actions in sepsis-induced cardiomyopathy, such as alleviating inflammation, inhibiting apoptosis, depressing oxidative damage, and repressing endoplasmic reticulum stress (Han et al., 2017). Some studies have also indicated that miR-22 inhibits breast cancer cell proliferation and suppress renal cell carcinoma by target sirt1 (Hariharan et al., 2011; Zhang S. et al., 2016; Zou et al., 2017). Nevertheless, the effect of miR-22/sirt1 signaling on sepsis-induced myocardial injury and cardiac dysfunction remains unclear.

Hence, in this study, we used a mouse CLP model and cardiac-specific miR-22 overexpression (miR-22^cOE^) and knockout (miR-22^cKO^) mice to verify the hypothesis that loss of miR-22 can protect the heart from sepsis and ameliorate cardiac dysfunction via the sirt1 signaling pathway.

## Materials and Methods

### Experimental Animals

The cardiac-specific knockout mice were generated by crossing miR-22-flox mice with αMHC-Cre mice (Huang et al., 2013). For miR-22 overexpression mice, portion of miR-22HG sequence containing miR-22 coding region were inserted into Rosa26 locus via homologous recombination to generate the miR-22-KI-flox mice (Supplementary Figure 1). MiR-22-KI-flox mice were then crossed with αMHC-Cre mice to generate the miR-22 cardiac-specific overexpression (miR-22^cOE^) mice. All experimental mice were 6–8 weeks old male mice and have free access to food and water. The littermates wild-type (WT) were used as the control group. The CLP mode**l** was established as previously described (Baker et al., 1983). The experimental mice were divided into (1) WT sham (sham), CLP, miR-22^cKO^, miR-22^cKO^ + CLP, (2) sham, CLP, miR-22^cOE^, and miR-22^cOE^ + CLP groups with 12 mice in every group.

### Cell Isolating and Treatment

Neonatal mice were used to isolate primary cardiomyocytes as previously described (Zhang et al., 2017). And then transfected cardiomyocytes with miR-22 negative control (NC), mimic (final concentration 5 μM) or inhibitor (final concentration 50 μM) according to the manufacturer’s instructions. Next, the cardiomyocytes were incubated with LPS for 12 h. Finally, the cardiomyocytes were divided into the following groups: (1) NC, NC + LPS, miR-22 inhibitor, miR-22 inhibitor + LPS, (2) NC, NC + LPS, miR-22 mimic, and miR-22 mimic + LPS.

### Western Blotting

Total protein was isolated from left ventricular (LV) tissue. LV tissue was separated from mice and homogenized by RIPA lysis buffer 8 h after CLP and mixed with protease inhibitor cocktail at a cocktail/RIPA ratio for 1:100. The protein concentration was quantified with a BCA kit (Thermo Fisher Scientific, TJ268882) to ensure equal total protein in each group. 10 or 12% SDS-PAGE was used to separate objective protein and the transferred protein to a 0.45 μm PVDF membrane (Millipore IPVH00010). Block the protein with 3% BSA for 2 h at room temperature. After that, incubate protein with primary antibodies at 4°C overnight and then the secondary antibodies. The Bio-Rad imaging system was used to obtain the images. All the experiments were repeated three times.

### TUNEL Staining and ATP Content Assay

The apoptosis of cardiomyocytes was detected by a TUNEL staining kit. An ATP Assay Kit (Sigma-Aldrich, MAK190) was used to measure the ATP content on the basis of the manufacturer’s instructions.

### Transmission Electron Microscope (TEM)

The autophagosomes and mitochondrial ultrastructure were observed by the TEM as previously described (Zhang et al., 2017). The cardiac tissue was cut into 2 mm cubes after washing with PBS to remove blood and fixed in 2% glutaraldehyde overnight at 4°C and the primary cardiomyocytes was translated into the polymerase chain reaction (PCR) tube and then centrifuged at 1,000 rpm for 5 min. The samples were prepared as previously described (Zhang et al., 2017). The typical autophagosomes are covered by a double membrane.

### Biochemical Analyses

Twenty-four hours after the operation, collected mice blood and then centrifuged samples at 3,000 g for 15 min. The LDH and CK-MB levels were quantified by the lactate dehydrogenase (LDH) assay kit (Sigma-Aldrich, MAK066) and creatine kinase-MB (CK-MB) assay kit (Sigma-Aldrich, MAK116).

Collecting and homogenizing myocardial tissue and IL-1β, IL-6, and TNFα were measured by the ELISA kits (Beyotime, P1301, P1326, and PT512).

### Echocardiogram

Echocardiography was measured by an M-mode echocardiography system with a 15 MHz linear transducer (Vevo 2100; Visual Sonics, Toronto, ON, Canada) as previously described (Shi et al., 2019). The mice were anesthetized with 2% isoflurane 24 h after CLP. Left ventricular ejection fraction (LVEF), left ventricular fractional shortening (LVFS), left ventricular end-diastolic diameter (LVEDD), and left ventricular end-systolic diameter (LVESD) were analyzed by using computer algorithms.

### Mitochondrial Membrane Potential (ΔΨ) Detection

A JC-1 assay kit (Beyotime, C2006) was used to evaluate ΔΨ of cardiomyocytes (Shi et al., 2019).

### Immunofluorescence

GFP-LC3 was detected by confocal microscopy as previously described (Zhang et al., 2017). The adenovirus GFP-LC3 was purchased from Hanbio Technology, Ltd. (Shanghai, China, MOI = 100).

### Hematoxylin-Eosin (HE) Staining

The heart tissues were separated from the experimental mice and fixed in 4% paraformaldehyde. The tissue was cut into 4–5 μm sections, Standard hematoxylin and eosin staining was performed following standard procedures. And then, the morphological damage of the tissues was observed by using a microscope.

### Statistical Analysis

All experiment data were analyzed by GraphPad Prism8 software. Results are presented as mean ± SEM. Unpaired 2-tailed Student’s *t*-test was performed when comparing two groups and one-way ANOVA were performed while compering multiple groups to calculate significance. The results were considered statistically significant while *P*-value < 0.05.

## Results

### Knocking out miR-22 Improves Cardiac Function and Ameliorate Myocardial Injury in CLP

**FIGURE 1:**
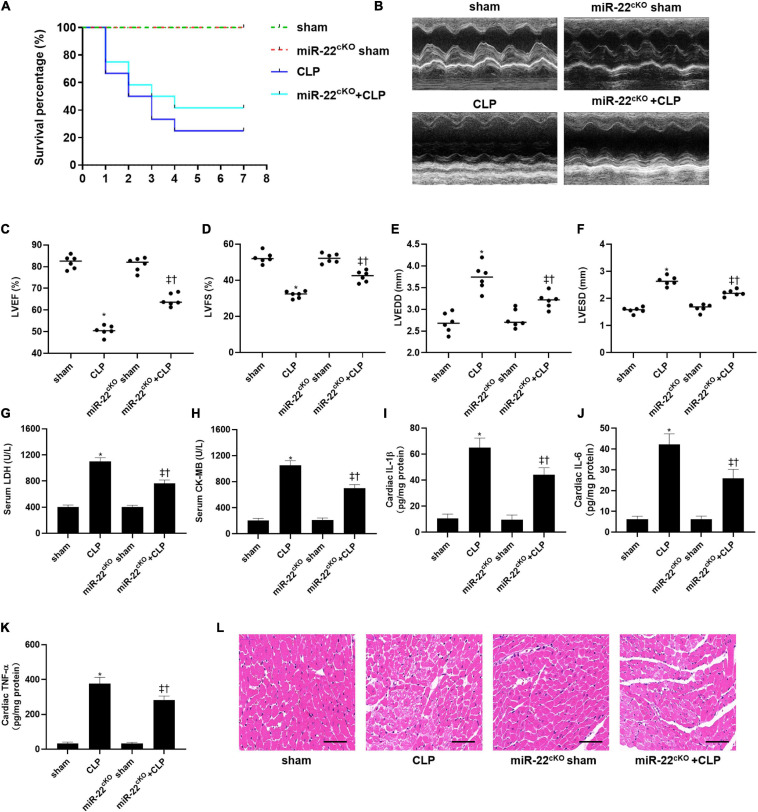
Knocking out miR-22 alleviates cardiac dysfunction and increases the survival rate in CLP-induced cardiomyopathy. **(A)** survival curves. Mortality was observed for 7 days, 12 mice of each group was used for comparison; **(B)** M-mode Echocardiograms representative image (*n* = 6); **(C)** LVEF; **(D)** LVFS; **(E)** LVESD; **(F)** LVEDD; **(G)** serum LDH (*n* = 6); **(H)** serum CK-MB (*n* = 6); **(I)** cardiac IL-1β (*n* = 6); **(J)** cardiac IL-6 (*n* = 6); **(K)** cardiac TNFα (*n* = 6); **(L)** HE staining representative images. Scale bar = 25 μm. Data were show as mean ± SEM. ^∗^*P* < 0.05 vs. sham group; ^‡^*P* < 0.05 vs. CLP group; ^†^*P* < 0.05 vs. miR-22^cKO^ sham group.

To explored the role of miR-22 in septic cardiomyopathy, the CLP model was established on the miR-22^cKO^ and wild type mice. The survival rate of the miR-22^cKO^ + CLP mice was also increased compared with the CLP mice after 7 days of observation (Figure 1A). Twenty-four hours after CLP, LVEF, and LVFS, markers of cardiac function, were obviously improved in miR-22^cKO^ + CLP mice. LVESD and LVEDD were decreased in the miR-22^cKO^ + CLP mice (Figures 1B–F). The results revealed that knocking out miR-22 can improve cardiac function. We next detected serum LDH and CK-MB, biomarkers of myocardial injury. Reduced levels of LDH and CK-MB indicated that knocking out miR-22 can alleviate myocardial injury (Figures 1G,H). Moreover, the cardiac IL-1β, IL-6, and TNFα content changed in line with LDH and CK-MB, and knocking out miR-22 decreased the IL-1β, IL-6, and TNFα content (Figures 1I–K). The HE staining results, the morphology of cardiomyocytes in the miR-22^cKO^ + CLP group was ameliorate compared with the CLP group, suggested that knocking out miR-22 can ameliorate myocardial injury in CLP-induced cardiomyopathy (Figure 1L).

### Inhibiting miR-22 Increases Autophagy via Sirt1 in CLP Induced Cardiomyopathy

**FIGURE 2:**
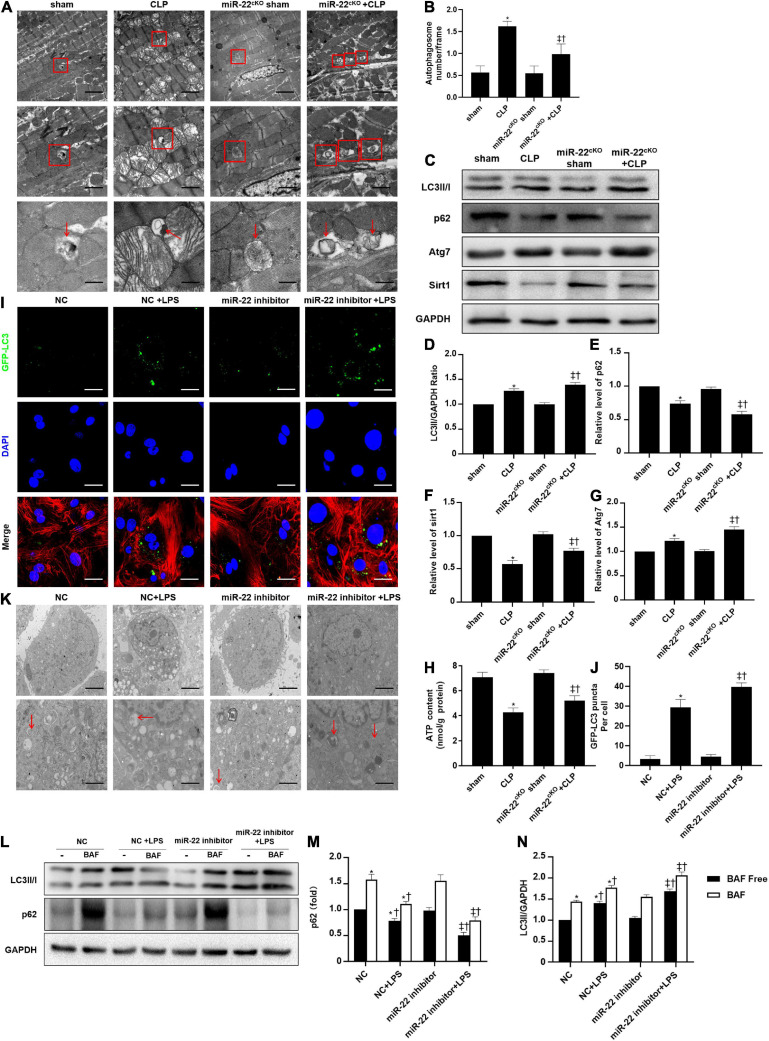
Knocking out miR-22 increases autophagy levels in CLP-induced cardiomyopathic myocardium and enhanced autophagic flux *in vitro* in the presence of LPS. **(A)** Representative images of myocardial mitochondria ultrastructural morphology underwent different treatments. **(B)** Quantity of autophagosomes. **(C)** Representative images of blots. **(D)** LC3-II/GAPDH ratio. **(E)** Relative p62 protein level ratio; **(F)** Relative sirt1 protein level ratio; **(G)** Relative Atg7 protein level ratio; Data were expressed as mean ± SEM. *P < 0.05 vs. sham group; ^‡^*P* < 0.05 vs. CLP group; ^†^*P* < 0.05 vs. miR-22^cKO^ sham group. **(H)** ATP content. **(I)** Representative images of mitochondria in neonatal mice cardiomyocytes; Scale bar = 2 μm, 1 μm, 500 nm. **(J,K)** Representative images and quantitative analysis of GFP-LC3 puncta; Scale bar = 20 μm. **(L–N)** Relative p62 protein level ratio with or without bafilomycin A1 treatment in condition of LPS; LC3-II/GAPDH ratio with or without bafilomycin A1 treatment in condition of LPS. *P < 0.05 vs. NC group; ^‡^*P* < 0.05 vs. NC + LPS group; ^†^*P* < 0.05 vs. miR-22 inhibitor group. All the experiments were repeated three times.

**FIGURE 3:**
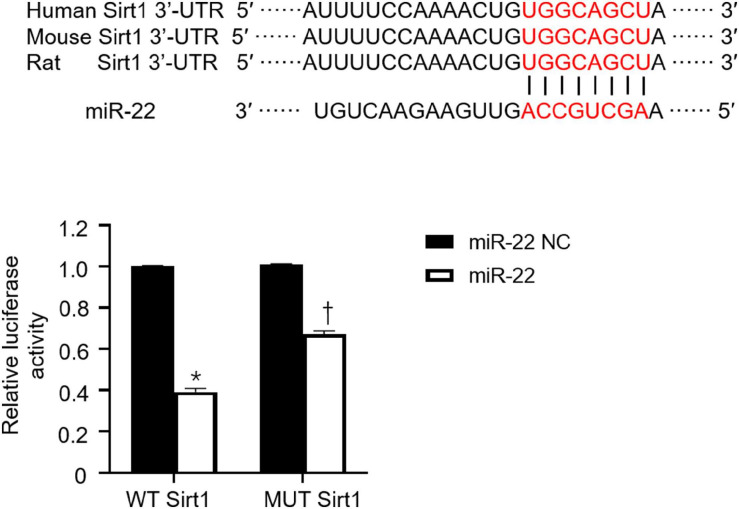
miR-22 exerted its effect during septic cardiomyopathy by targeting sirt1. Results of luciferase report. *P < 0.05 vs. NC group in sirt1 group; ^†^*P* < 0.05 vs. NC group in MUT sirt1 group. All the experiments were repeated three times.

To identify the cardioprotective effect of knocking out miR-22, we determined the number of autophagosomes and mitochondrial morphology by TEM and found that inhibiting miR-22 can increase autophagosomes and maintain mitochondrial morphology in the CLP mice compared with the sham mice and in the miR-22^cKO^ + CLP mice compared with the CLP mice (Figures 2A,B). Western blotting results suggested that the LC3II/GAPDH ratio was higher in the miR-22^cKO^ + CLP mice than that in the CLP mice and increased in the CLP mice compared with the sham mice (Figures 2C,D). P62 expression in the CLP group was decreased compared with that in the sham group and higher than that in the miR-22^cKO^ + CLP group (Figure 2E). The sirt1 and ATG7 expression levels are shown in Figures 2F,G. And sirt1 mRNA level and sirt1 enzyme activity have been shown in Supplementary Figures 5A,B. The results suggested that knocking out miR-22 can promote autophagy and that miR-22 may target sirt1 to alter the autophagy levels. The autophagy level altered over time as shown in Supplementary Figures 6A,B. In addition, the ATP content changed in line with LDH and CK-MB, and knocking out miR-22 increased the ATP content compared with the CLP group (Figure 2H). Next, we transfected adenovirus GFP-LC3 into cardiomyocytes and stained nuclei with DAPI. As shown in Figures 2I,J, there was a higher number of green puncta in the NC + LPS group than in the NC group, and the number of green puncta in the miR-22 inhibitor + LPS group was higher than that in the NC + LPS group. In addition, the TEM results revealed that inhibiting miR-22 may mitigate mitochondrial damage by observing the morphology of mitochondria (Figure 2K) and increase the ΔΨ in LPS treated cardiomyocytes, as confirmed by a JC-1 assay kit (Supplementary Figure 2). Inhibiting miR-22 also enhanced autophagic flux, as confirmed by increased LC3II/GAPDH ratio and reduced p62 expression under the condition of a lysosomal inhibitor bafilomycin A1 (Figures 2L–N). It has been reported that miR-22 can target sirt1 to exert it effects (Huang et al., 2013). Finally, we confirmed that sirt1 is the target of miR-22 in septic cardiomyopathy by the luciferase reporter assay (Figure 3).

### Knocking Out miR-22 Ameliorates Cardiomyocyte Apoptosis in CLP

**FIGURE 4:**
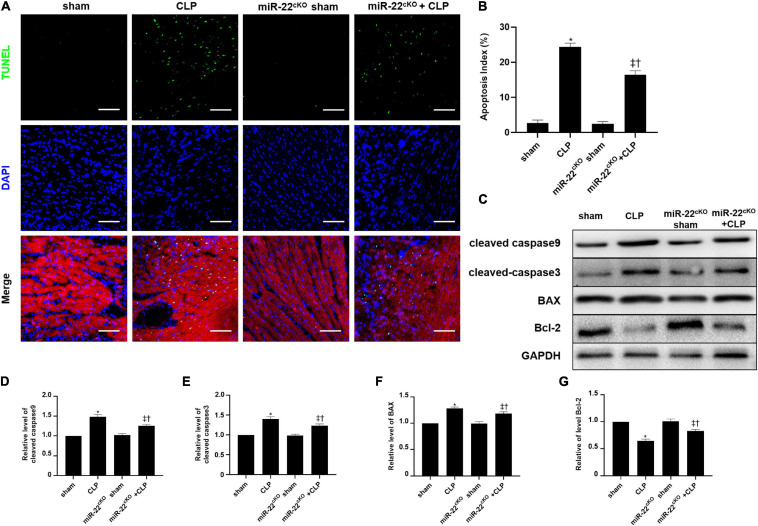
Knocking out miR-22 reduces the apoptotic index of the myocardium after CLP and alleviates myocardial injury. **(A)** Representative images of TUNEL staining (myocardium); Scale bar = 50 μm. **(B)** Percentage of TUNEL-positive nuclei. **(C)** Representative Western blots. **(D)** Relative cleaved-caspase9 protein level ratio. **(E)** Relative cleaved-caspase3 protein level ratio. **(F)** Relative BAX protein level ratio. **(G)** Relative Bcl-2 protein level ratio. Data were expressed as mean ± SEM. ^∗^*P* < 0.05 vs. sham group; ^‡^*P* < 0.05 vs. CLP group; ^†^*P* < 0.05 vs. miR-22^cKO^ sham group. **(G)** Representative images of TUNEL staining; Scale bar = 25 μm; **(H)** Percentage of TUNEL-positive nuclei. ^∗^*P* < 0.05 vs. NC group; ^‡^*P* < 0.05 vs. NC + LPS group; ^†^*P* < 0.05 vs. miR-22 inhibitor group. All the experiments were repeated three times.

The frozen sections and paraffin sections to perform TUNEL staining 24 h after CLP. *In vivo*, the myocardial cell apoptotic index in the miR-22^cKO^ + CLP mice was obviously decreased compared with that in the CLP group (Figures 4A,B). We also measured the apoptotic proteins collected from LV tissue in every group, the levels of BAX, cleaved-caspase 3, and cleaved-caspase 9 were decreased by knocking out miR-22 subjected to CLP, while Bcl-2 was increased (Figures 4C–G). Finally, a rescue experiment was designed to identify that miR-22 plays its effect by targeting sirt1. While knocking down sirt1, the increased expression of cleaved-caspase9, cleaved-caspase3 and BAX while decreased bcl-2 level suggested an increased apoptosis. And increased expression level of p62 and decreased ratio of LC3II/GAPDH revealed that the level of autophagy was decreased. The results were displayed in Supplementary Figure 3.

### Overexpressing miR-22 Aggravates Cardiac Function and Myocardial Injury in CLP

**FIGURE 5:**
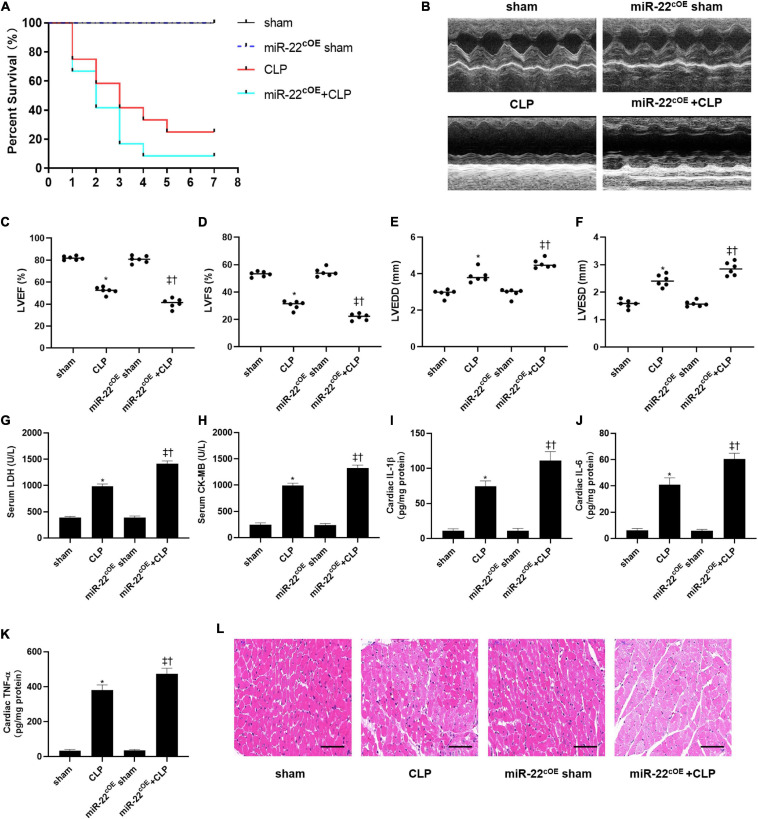
Overexpressing miR-22 aggravates cardiac function and reduces the survival rate in CLP-induced cardiomyopathy. **(A)** Survival curves. Mortality was observed for 7 days, 12 mice of each group was used for comparison. **(B)** Representative images of M-mode Echocardiograms (*n* = 6); **(C)** LVEF; **(D)** LVFS; **(E)** LVESD; **(F)** LVEDD; **(G)** serum LDH (*n* = 6); **(H)** serum CK-MB (*n* = 6); **(I)** cardiac IL-1β (*n* = 6); **(J)** cardiac IL-6 (*n* = 6); **(K)** cardiac TNFα (*n* = 6); **(L)** HE staining representative images. Scale bar = 25 μm. Data were expressed as mean ± SEM.^∗^*P* < 0.05 vs. sham group; ^‡^*P* < 0.05 vs. CLP group; ^†^*P* < 0.05 vs. miR-22^cOE^ sham group. All the experiments were repeated three times.

We used cardiac-specific knock-in mice to verify our hypothesis along with gain of function analyses. We observed the survival of every group (*n* = 12) for 7 days, and the results are shown in Figure 5A. LVEF and LVFS were significantly decreased in miR-22^cOE^ + CLP mice and LVEDD and LVESD were increased compared with CLP mice (Figures 5B–F). Our data indicated that overexpressing miR-22 in CLP mice obviously increased LDH and CK-MB level compared with the CLP mice (Figures 5G,H). Then, IL-1β, IL-6, and TNFα was measured by the Elisa kits. There was a significant increase in the miR-22^cOE^ + CLP group compared with the CLP group (Figures 5I–K). Finally, HE staining revealed that myocardial damage was aggravated when miR-22 was overexpressed (Figure 5L).

### miR-22 Inhibits Autophagy via Sirt1 in CLP Induced Cardiomyopathy

**FIGURE 6:**
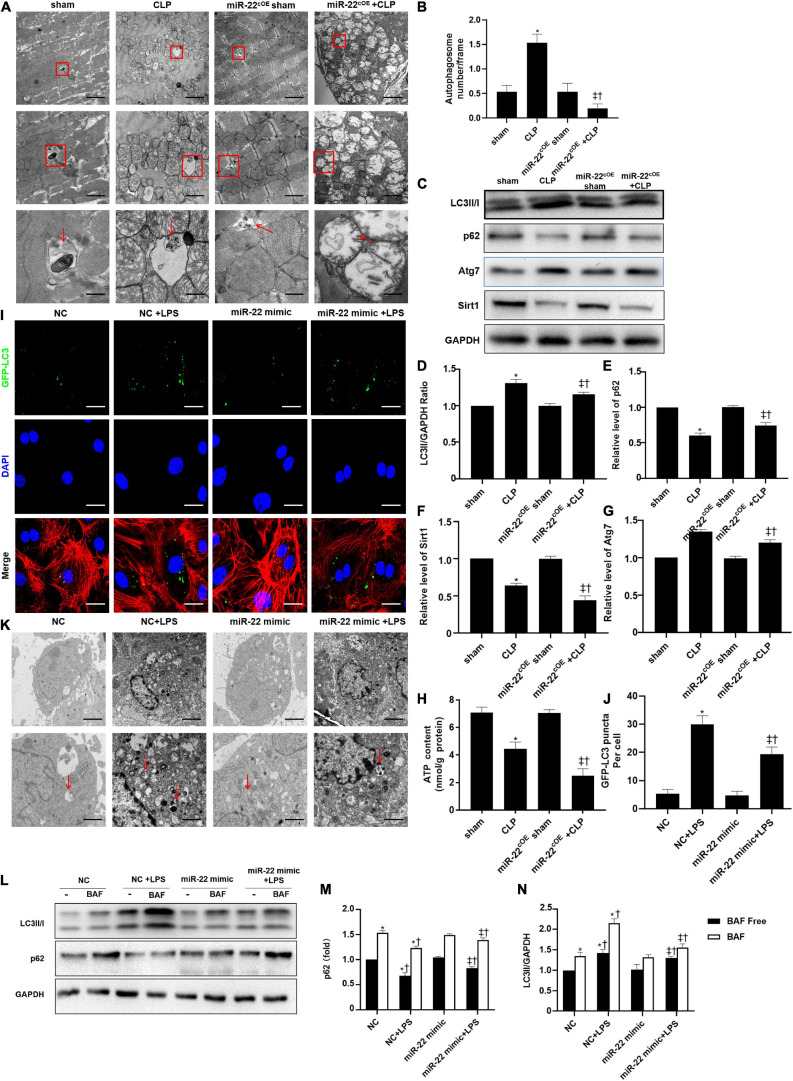
Overexpressing miR-22 reduces autophagy levels in CLP-induced cardiomyopathic myocardium and inhibits autophagic flux *in vitro* in the presence of LPS. **(A)** Representative images of myocardial mitochondria ultrastructural morphology that underwent different treatments. **(B)** Quantity of autophagosomes. **(C)** Representative images of blots. **(D)** LC3-II/GAPDH ratio. **(E)** Relative p62 protein level ratio. **(F)** Relative sirt1 protein level ratio. **(G)** Relative Atg7 protein level ratio; Data were expressed as mean ± SEM. ^∗^*P* < 0.05 vs. sham group; ^‡^*P* < 0.05 vs. CLP group; ^†^*P* < 0.05 vs. miR-22^cOE^ sham group. **(H)** ATP content (*n* = 6). **(I,J)** Representative images and quantitative analysis of GFP-LC3 puncta; Scale bar = 20 μm. **(K)** Representative images of mitochondria in neonatal mice cardiomyocytes. **(L–N)** Relative p62 protein level ratio with or without bafilomycin A1 treatment in condition of LPS; LC3-II/GAPDH ratio with or without bafilomycin A1 treatment in condition of LPS. ^∗^*P* < 0.05 vs. NC group; ^‡^*P* < 0.05 vs. NC + LPS group; ^†^*P* < 0.05 vs. miR-22 mimic group. All the experiments were repeated three times.

The TEM results suggested that overexpressing miR-22 may aggravate mitochondrial damage by observing the morphology of the mitochondria (Figures 6A,B). Next, western blotting data revealed that LC3II/GAPDH ratio and ATG7 expression were decreased when miR-22 was overexpressed compared with the CLP group (Figures 6C–G). The p62 level rose in the miR-22^cOE^ + CLP mice compared with that in the CLP group, and sirt1 expression is shown in Figures 6C–G. sirt1 mRNA level and sirt1 enzyme activity have been shown in Supplementary Figures 7A,B. The results suggested that miR-22 can inhibit autophagy and may decrease sirt1 to inhibit the autophagy levels. ATP content was significantly decreased in miR-22^cOE^ + CLP mice compared with CLP mice (Figure 6H). Then, we performed immunofluorescence experiments to observe autophagic flux by using confocal microscopy (Figure 6I). As shown in Figure 6J, there was a lower number of green puncta in the miR-22 mimic + LPS group than the LPS group. Moreover, we observed the number of autophagosomes by TEM, and the data indicated that reduction of autophagosomes in the miR-22 mimic + LPS group compared with the NC + LPS group (Figure 6K). In addition, JC-1 fluorescence imaging suggested that overexpressing miR-22 decreased the ΔΨ in cardiomyocytes under the condition of LPS (Supplementary Figure 4). Overexpressing miR-22 also suppresses autophagic flux, confirmed by the decreased LC3II/GAPDH ratio and increased p62 level under bafilomycin A1 (Figures 6L–N).

### Overexpressing miR-22 Increases Cardiomyocyte Apoptosis in CLP

**FIGURE 7:**
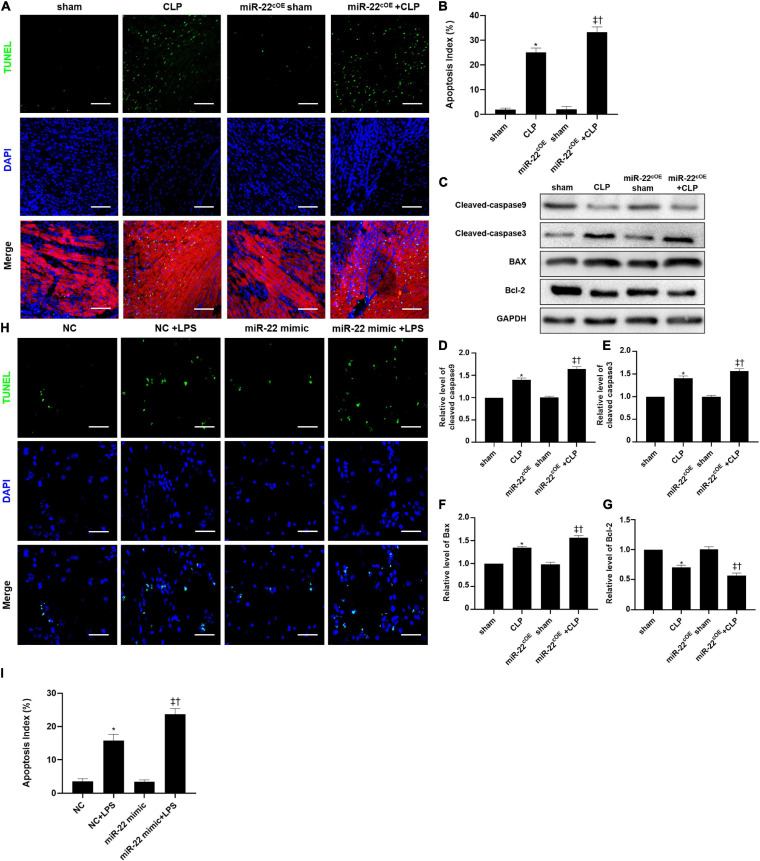
**(A)** Representative images of TUNEL staining (myocardium); Scale bar = 50 μm. **(B)** Percentage of TUNEL-positive nuclei. **(C)** Representative Western blots. **(D)** Relative cleaved-caspase9 protein level ratio. **(E)** Relative cleaved-caspase3 protein level ratio. **(F)** Relative BAX protein level ratio. **(G)** Relative Bcl-2 protein level ratio; Data were expressed as mean ± SEM. ^∗^*P* < 0.05 vs. sham group; ^‡^*P* < 0.05 vs. CLP group; ^†^*P* < 0.05 vs. miR-22^cOE^ sham group. **(H)** Representative TUNEL staining images; Scale bar = 50 μm. **(I)** Percentage of TUNEL-positive nuclei. ^∗^*P* < 0.05 vs. NC group; ^‡^*P* < 0.05 vs. NC + LPS group; ^†^*P* < 0.05 vs. miR-22 mimic group. All the experiments were repeated three times.

Overexpressing miR-22 aggravated the apoptotic index compared with that of the CLP mice, as shown by TUNEL assays (Figures 7A,B). Concomitantly, the levels of cleaved-caspase9, cleaved-caspase3, and BAX in the miR-22^cOE^ + CLP mice were higher than those in the CLP mice, while the bcl-2 expression was reduced (Figures 7C–G). *In vitro*, we used a miR-22 mimic to overexpress miR-22 and LPS to establish a cardiomyopathy model. The results were consistent with the *in vivo* CLP-induced cardiomyopathy results (Figures 7H,I).

## Discussion

Septic cardiomyopathy is a common complication caused by sepsis, which occurs in approximately 25% of septic patients (Dombrovskiy et al., 2007). Septic cardiomyopathy occurs in patients with ventricular dilatation, decreased ventricular contractility and cardiac dysfunction (Takasu et al., 2013; Martin et al., 2019). However, there are no specific therapeutics for septic cardiomyopathy. The present study indicated that miR-22 promoted the progression of septic cardiomyopathy as evidenced by aggravated cardiac dysfunction. Melatonin may be a new strategy for the treatment of septic cardiomyopathy. It has been reported that the main manifestations of sepsis are ventricular dilatation, decreased ejection fraction and systolic dysfunction (Wagner et al., 2015; Han et al., 2017). In our study, LVEF, LVFS indicated a significant reduction in cardiac function. In addition, the increased levels of LDH and CK-MB proved that myocardial injury was aggravated. HE staining further confirmed our hypothesis that knocking out miR-22 can improve cardiac function, but overexpressing miR-22 aggravated it.

Autophagy is a highly conserved process in eukaryotes. By degrading damaged cellular components to ensure cellular homeostasis, this process regulates cell quality and provides energy for the cell (Zech et al., 2019). Autophagy plays a crucial role in maintaining cardiomyocyte stability (Zhang M. et al., 2016; Feidantsis et al., 2018; Pei et al., 2018; Shi et al., 2019). A recent study revealed that beclin-1-dependent autophagy exert a cardioprotective effect the in LPS-induced septic cardiomyopathy (Sun et al., 2018). Another study also proved that inhibiting the mTOR pathway can protect the heart from sepsis in a CLP rat model by increasing autophagy (Han et al., 2018). MiR-22 was reported to regulate autophagy in diabetic nephropathy by targeting PTEN (Zhang et al., 2018) and p38α in cardiomyocytes (Li et al., 2016). Another study showed that miR-22 inhibited autophagy by the Notch signaling pathway in human ovarian cancer cells (Li et al., 2018). Moreover, we observed that inhibiting miR-22 can upregulate autophagy levels, while the level of sirt1 was increased. The autophagy level was upregulated by LC3, p62, and Atg7 expression, but sirt1 was downregulated in the CLP group, revealing that autophagy may show a compensatory increase. In the present study, overexpressed miR-22 inhibited autophagy, as identified by the increased p62 expression and decreased LC3II/GAPDH ratio. We also verified these results through a loss-of-function experiment. Our results suggested that knocking out miR-22 exerts a cardioprotective effect in septic cardiomyopathy by increasing autophagy levels.

Numerous studies have reported that sirt1 plays an antiapoptotic role in the development of cardiomyocyte injury (Huang et al., 2019; Jiang et al., 2019; Wu et al., 2019; Ying et al., 2019; Zhang et al., 2019). Apoptosis takes an important part in cardiac dysfunction when the heart is exposed to stress or injury. Cardiac function was improved when the level of sirt1 was upregulated. Previous studies have revealed the protective effect of sirt1 activation in sepsis. And upregulating sirt1 promotes CLP-induced cardiomyopathy, which benefits from a decrease in apoptosis and ER stress (Han et al., 2017). Another study demonstrated that melatonin upregulated sirt1 to protect the heart from sepsis by regulating apoptosis and autophagy (Zhang et al., 2019). miR-22 is involved in cardiomyopathy by targeting sirt1 (Tang et al., 2018; Xu et al., 2020), however, the role of miR-22 in cardiomyopathy remains controversial. It has been reported that inhibiting miR-22 prevents doxorubicin-induced cardiotoxicity by upregulating sirt1 (Xu et al., 2020). However, in another study, overexpressing miR-22 attenuated oxidative stress via sirt1 (Tang et al., 2018). In the present study, knocking out miR-22 reduced myocardial injury, as evidenced by the reduced apoptotic index of cardiomyocytes and the decreased expression of apoptotic proteins, including cleaved-caspase3, cleaved caspase9 and BAX. In addition, the luciferase reporter assay suggested that miR-22 exerts a cardioprotective role in CLP-induced septic cardiomyopathy by targeting sirt1. A study has reported that cell death is rare in sepsis-induced cardiac dysfunction, but focal mitochondrial injury does occur [1]. However, another study reported that apoptosis proteins were increased in sepsis mice heart and rapamycin can exert a cardiac protective effect by increased ATP levels, and decreased inflammatory responses, as well as decreased cardiomyocyte death in the left ventricle [2].

**FIGURE 8:**
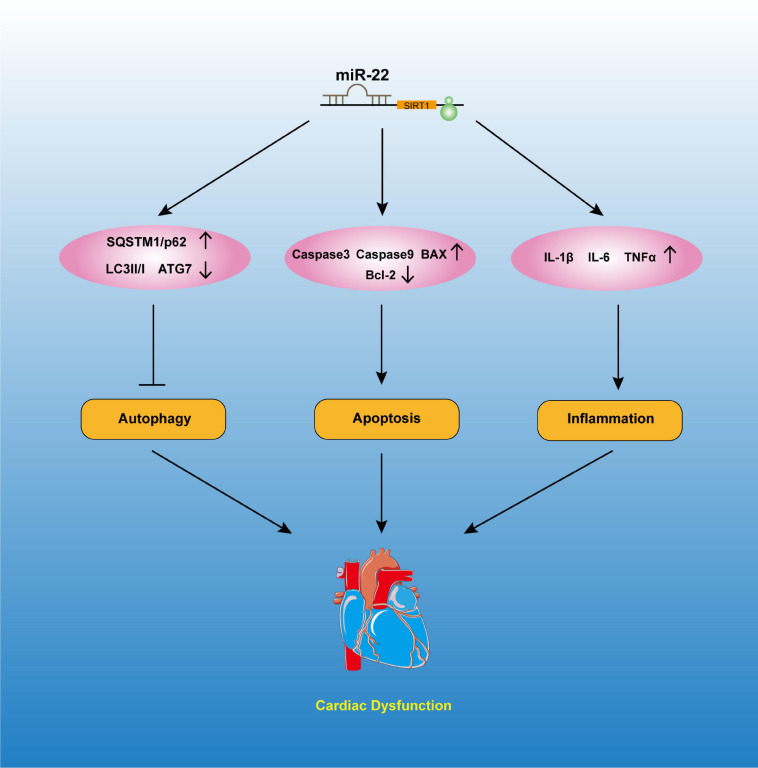
The possible mechanisms involved in the effects of miR-22 in septic cardiomyopathy.

Mitochondria is vital for maintaining the ventricle contractile function of the heart. Mitochondria are the most important ATP delivery system for the heart, and mitochondrial dysfunction is pernicious to the heart. Studies reported that mitochondrial dysfunction can significantly lead to cardiac dysfunction, such as I/R injury, DM and septic cardiomyopathy. A study revealed edema of the mitochondrial matrix in septic patients’ hearts (Cimolai et al., 2015). We also observed more vacuolized mitochondria in the mice that were subjected to a CLP operation than in those subjected to the sham operation. Knocking out miR-22 can reduce the number of vacuolized mitochondria, while overexpressing miR-22 can increase it. Mitochondrial dysfunction can be measured by ATP content and membrane potential. In the present study, our data suggested that knocking out miR-22 reduced mitochondrial membrane potential, as proven by a JC-1 assay, and increased ATP content during sepsis. These results indicated that miR-22 may aggravate mitochondrial dysfunction. The data suggested inhibiting miR-22 mediated physiological process may exert a protective mechanism against sepsis induced cardiomyopathy (Figure 8).

## Conclusion

In conclusion, our results revealed that inhibiting miR-22 can improve cardiac function, reduce cardiomyocyte apoptosis and upregulate autophagy in CLP-induced cardiomyopathy. The protective effect of inhibiting miR-22 was confirmed by targeting sirt1 signaling. The present study provides a new direction for septic cardiomyopathy, which is vital for the treatment of septic cardiomyopathy.

## Data Availability Statement

The original contributions presented in the study are included in the article/Supplementary Material, further inquiries can be directed to the corresponding author/s.

## Ethics Statement

The animal study was reviewed and approved by the Guide for the Care and Use of Laboratory Animals and the Guidelines for the Welfare of Experimental Animals issued by the Ethics Committee on Animal Care of the Fourth Military Medical University.

## Author Contributions

MZ, ZH, and YL participated in the conception and design of the study. RW, YX, WZ, and YF were responsible for the analysis and interpretation of data. TY, DZ, JL, TW, and HZ contributed to drafting the article. All authors have read and approved the final submitted manuscript.

## Conflict of Interest

The authors declare that the research was conducted in the absence of any commercial or financial relationships that could be construed as a potential conflict of interest.
