# Supplementary material for: Inhibiting miR-22 Alleviates Cardiac Dysfunction by Regulating Sirt1 in Septic Cardiomyopathy
Source: Front Cell Dev Biol. 2021 Apr 1;9:650666. doi: 10.3389/fcell.2021.650666 (PMC8047209; doi:10.3389/fcell.2021.650666)
Supplement: Supplementary file 1 [file Data_Sheet_1.docx]

**Supplemental Data**

**Agents**

The following antibodies were used: p62 (1:1000; Abcam, ab91526), cleaved caspase-3 (1:1000; Abcam, ab2302), cleaved caspase-9 (1:1000; Abcam, ab2324), Bcl-2 (1:1000; Abcam, ab32124), BAX (1:1000, Abcam, ab32503), Sirt1 (1:1000, Cell Signaling, 8469), LC3A/B (1:1000, Cell Signaling, 12741), Atg7 (1:1000, Cell Signaling, 8558), GAPDH (1:500, Santa Cruz, CA, USA) and secondary antibodies (anti-mouse/rabbit IgG) conjugated with horseradish peroxidase (1:5000, Cell Signaling). Terminal-deoxynucleotidyl transferase-mediated nick end labeling (TUNEL) staining kits and 4′,6-diamino-2-phenylindole (DAPI) were purchased from Sigma-Aldrich (St. Louis, MO, USA). Sirt1 siRNA was purchased from Santa Cruz (CA, USA).

**Supplemental Figure and Figure Legends**

**Figure1**

**
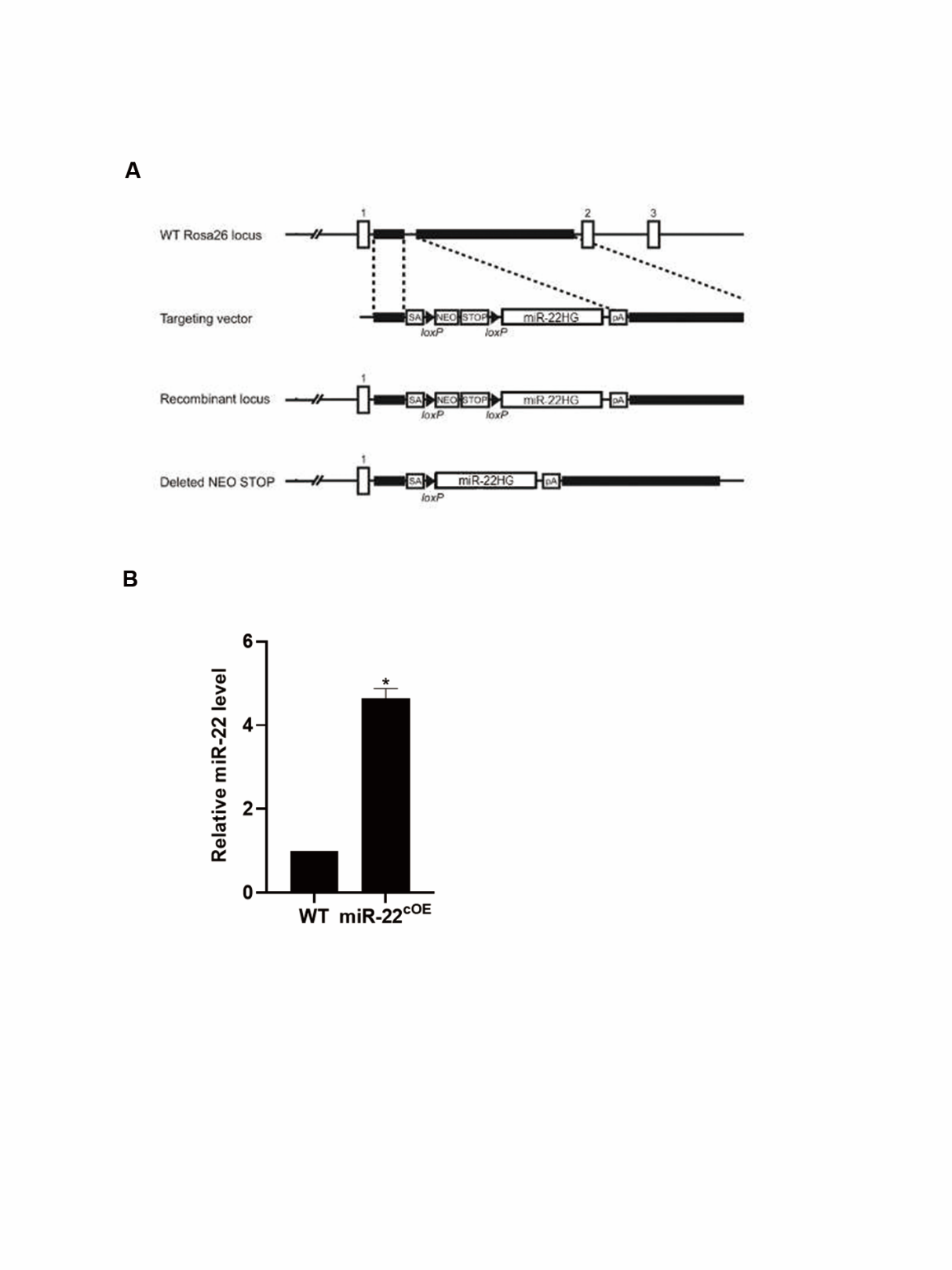
**

**Supplement Figure1. Generation of miR-22 mutant mice.**

**Figure2**

**
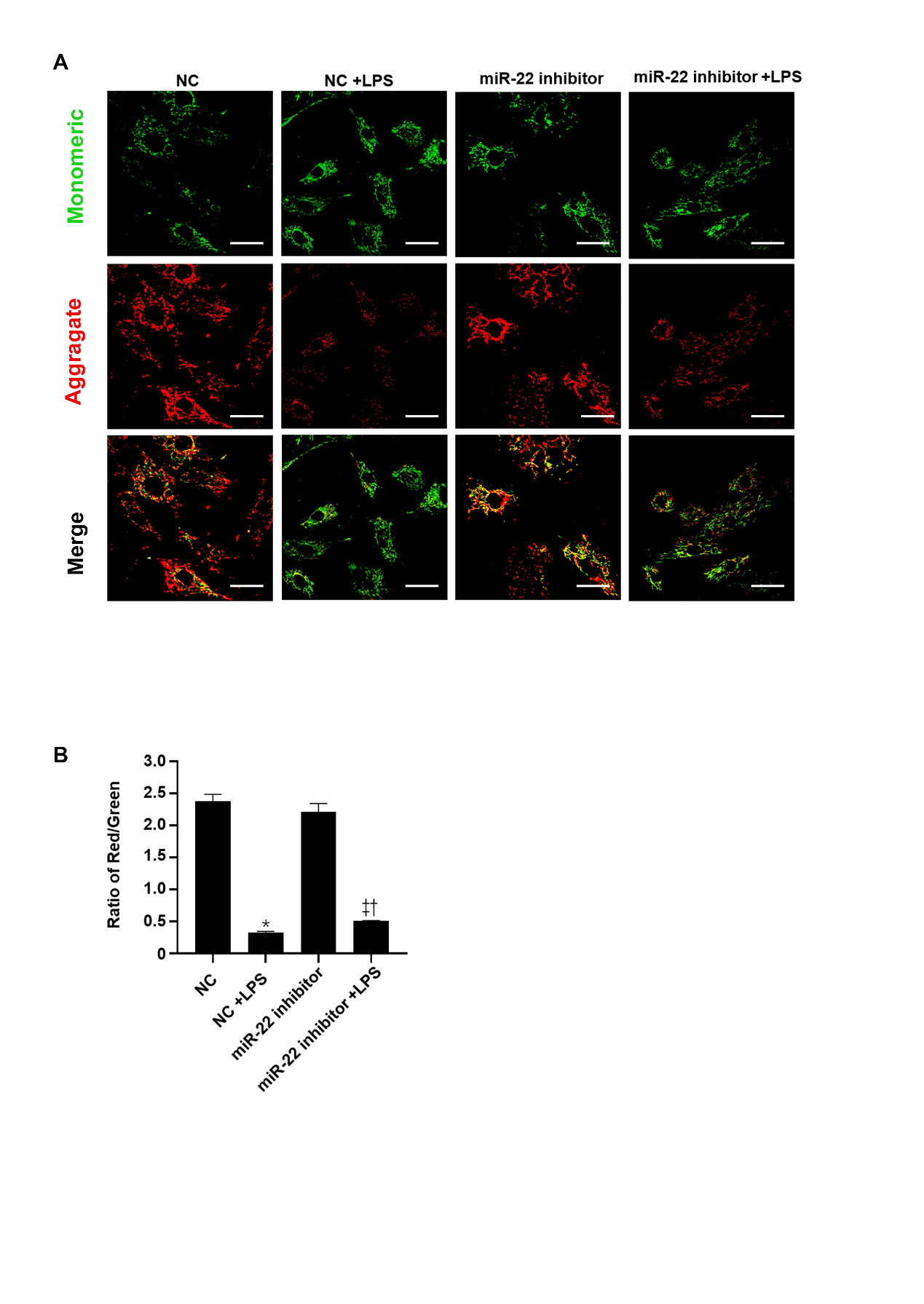
**

**Supplement Figure2 Inhibiting miR-22 improved mitochondrial membrane potential.**

1. Representative images of JC-1 B. The ratio of aggregated(red) and monomeric (green) JC-1. The columns and error bars represent the means and SEM. *P < 0.05 vs miR-22 NC; †P < 0.05 vs miR-22 NC +LPS; ‡P < 0.05 vs miR-22 inhibitor.

**Figure3**

**
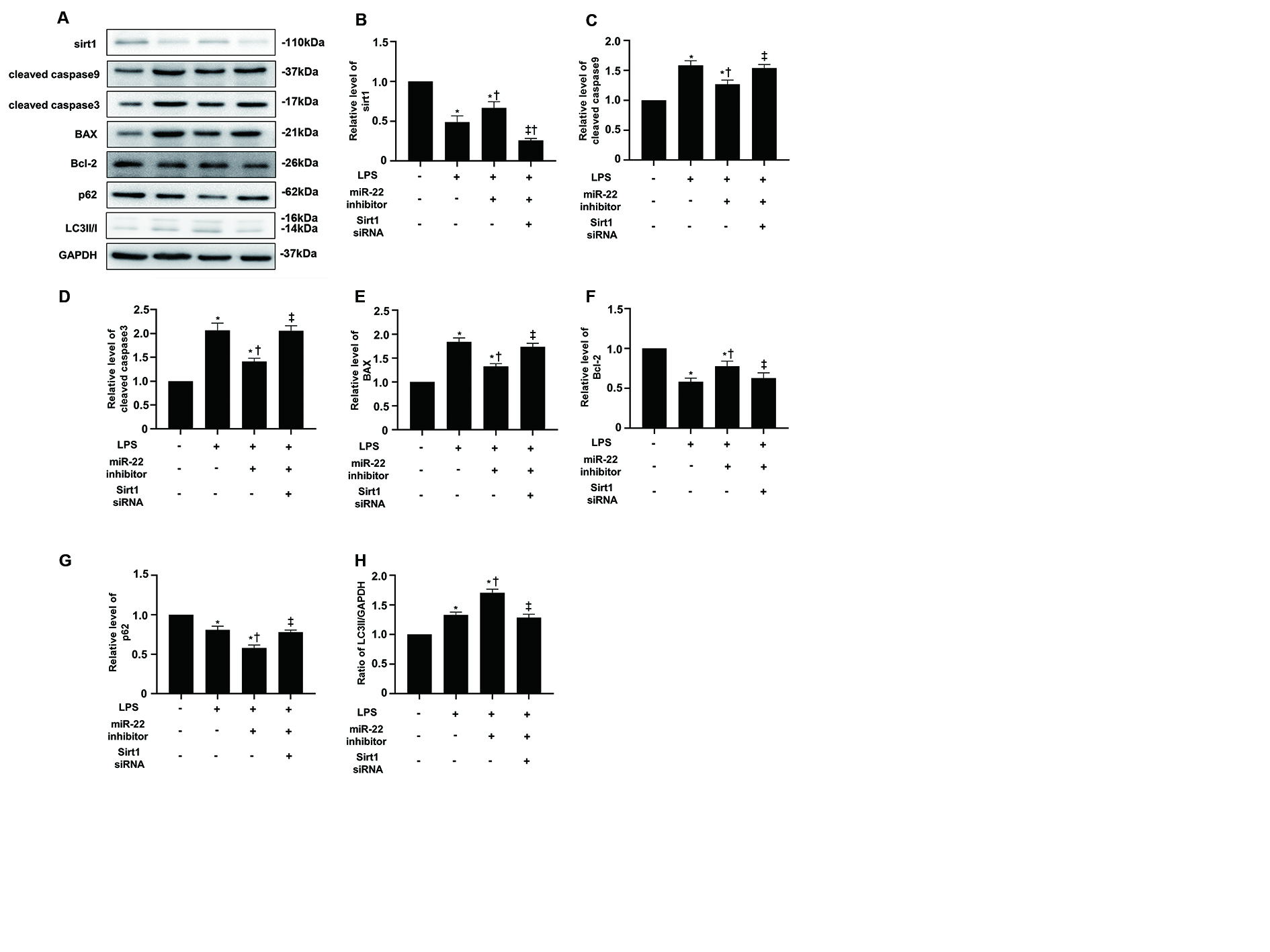
**

**Supplement Figure3 inhibiting miR-22 plays a cardioprotective effect by targeting sirt1.**

1. The representative western blot images; B. Relative protein level of sirt1; C. Relative protein level of cleaved caspase9; D. Relative protein level of cleaved caspase3; E. Relative protein level of BAX; F. Relative protein level of Bcl-2; G. Relative protein level of p62; H. Ratio of LC3II/GAPDH. *P < 0.05 vs NC; †P < 0.05 vs LPS; ‡P < 0.05 vs miR-22 inhibitor +LPS.

**Figure4**

**
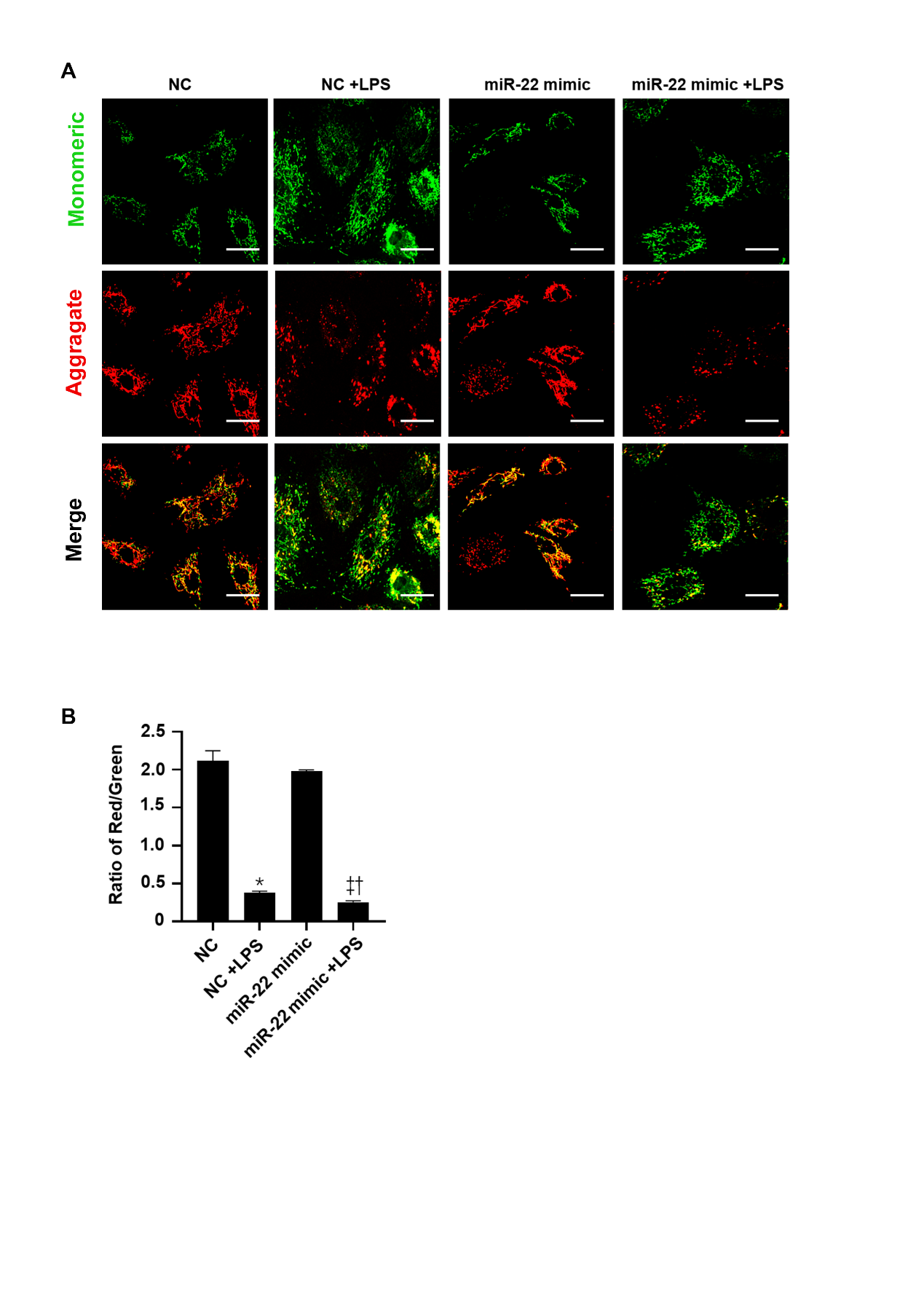
**

**Supplement Figure4 Overexpressing miR-22 decreased mitochondrial membrane potential.**

A. Representative images of JC-1 B. The ratio of aggregated(red) and monomeric (green) JC-1. The columns and error bars represent the means and SEM. *P < 0.05 vs miR-22 NC; †P < 0.05 vs miR-22 NC +LPS; ‡P < 0.05 vs miR-22 mimic.

**Figure5**

B

A

**Supplement Figure5 Sirt1 mRNA level and sirt1 enzyme activity of miR-22 knocking out mice.**

A. Relative sirt1 mRNA expression; B. sirt1 enzyme activity. The columns and error bars represent the means and SEM. *P < 0.05 vs sham; †P < 0.05 vs CLP; ‡P < 0.05 vs miR-22^cKO^.

Figure6


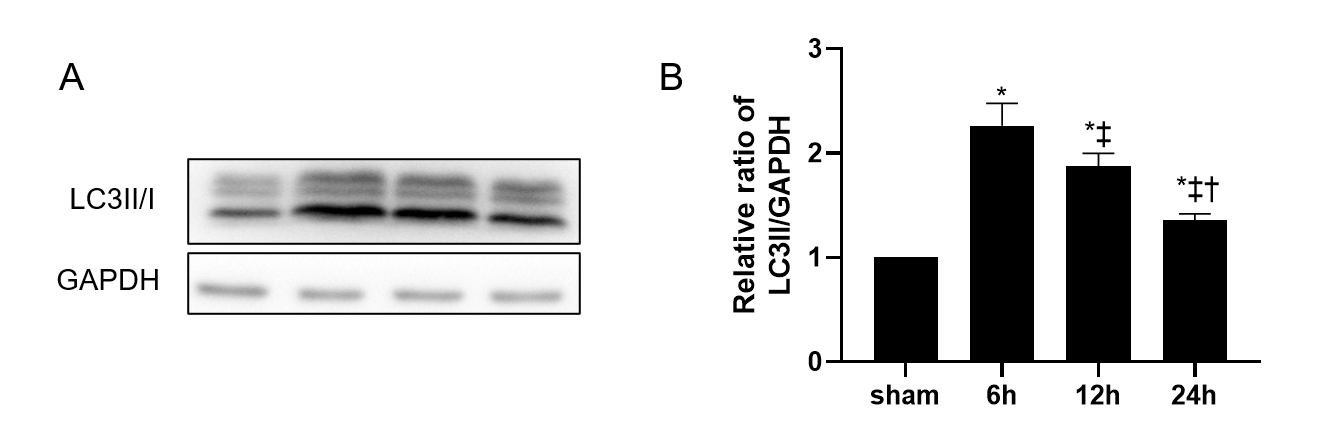


**Supplement Figure6 LC3I/II expression altered over time after CLP.**

A. Representative western blot image of LC3I/II after CLP; B. Ratio of LC3II/GAPDH The columns and error bars represent the means and SEM. *P < 0.05 vs sham; †P < 0.05 vs 6h post CLP; ‡P < 0.05 vs 12h post CLP.

**Figure7**

A

B

**Supplement Figure7 Sirt1 mRNA level and sirt1 enzyme activity of miR-22 overexpressing mice.**

A. Relative sirt1 mRNA expression; B. sirt1 enzyme activity. The columns and error bars represent the means and SEM. *P < 0.05 vs sham; †P < 0.05 vs CLP; ‡P < 0.05 vs miR-22^cOE^.
